# Distance in Information and Statistical Physics III

**DOI:** 10.3390/e25010110

**Published:** 2023-01-05

**Authors:** Takuya Yamano

**Affiliations:** Faculty of Science, Kanagawa University, 2946, 6-233 Tsuchiya, Hiratsuka 259-1293, Japan; yamano@amy.hi-ho.ne.jp

This Special Issue is a subsequent edition of a previous collection that focused on the notion of distance in two major fields: Distance in Information and Statistical Physics Volume 2 [1]. Depending on the disciplines, the term “distance” can be used in a varied sense, as it can be used in relation to geometry, divergence, relative entropy, discrimination, degrees of irreversibility, the arrow of time, and more. Studying the properties of these fundamental measures and identifying connections among them are of key interest and great value.

It is no exaggeration to say that distance measures and changes in them in time and space make the description of systems possible. In particular, information sciences and statistical physics benefit from various divergence measures or relative entropy. Identifying and measuring the closeness of distinguishable distributions are also significant processes in the functioning of machine learning in AI.

This Special Issue compiles five quality contributions from researchers whose backgrounds are in mathematics, physics, and information science. Of the planned keywords listed in this Special Issue [2], *f*-divergence, differential entropy, Kullback–Leibler divergence, Jensen–Shannon divergence, Jeffreys divergence, Fisher information, and others are covered in the contributing papers.

Contreras-Reyes [3] considers the two methods for the location parameter estimation of skew-normal distribution: the least square estimator and the best unbiased estimator. In this process, the author presents two lower bounds for differential entropy and obtains both the lower and upper bounds for the Fisher information of the location parameter. The behavior of these bounds is numerically illustrated.

Entropic power inequality is extremely versatile. In the paper written by Wang, Stavrou, and Skoglund [4], the authors derive two new generalizations of Talagrand-type inequalities, which are well-known in optimal transport theory. With these inequalities, the geometry implied by Sinkhorn distance is shown to be smoothed out in the sense of measure concentration. Since Sinkhorn distance is a generalization of Wasserstein distance with an extra entropic constraint, the quantification of the extra cost is needed. Numerical simulations confirmed these results.

Among many types of *f*-divergence, Kluza [5] introduces Jensen–Sharma–Mittal and Jeffreys–Sharma–Mittal divergences and shows their properties, including the lower and upper bounds. These divergences are extensions of Sharma–Mittal-type divergences, in which two parameters are contained, and they are the generalizations of Rényi, Tsallis, and Kullback–Leibler types with suitable choices of divergence functions.

Nielsen [6] proposes a simple and fast heuristic method to approximate the Jeffreys divergence between two univariate Gaussian mixtures with an arbitrary number of components. The measurement of the goodness of fit between a Gaussian mixture and an exponential polynomial density is attempted using a generalization of relative Fisher information. A numerical demonstration of a considerable improvement in computational time is reported for the proposed approximation under particular circumstances.

Fisher information has also been used to quantify the information of wave functions for quantum mechanical systems, such as hydrogen-like atoms. Paper [7] investigates the Fisher information and relative Fisher information of the radial wave functions of the free-electron Landau states realized by electrons in a uniform magnetic field. With numerical evaluation of the generalized Laguerre polynomials, the study reveals that these measures of information change monotonically with quantum numbers.

I believe these papers stand out in their respective disciplines and will inspire future research in the various scientific communities.
